# Targeted gene panel provides advantages over whole-exome sequencing for diagnosing obesity and diabetes mellitus

**DOI:** 10.1093/jmcb/mjad040

**Published:** 2023-06-16

**Authors:** Hairong Yu, Haoyong Yu, Rong Zhang, Danfeng Peng, Dandan Yan, Yunjuan Gu, Yuqian Bao, Weiping Jia, Hong Zhang, Cheng Hu

**Affiliations:** Shanghai Diabetes Institute, Shanghai Key Laboratory of Diabetes Mellitus, Shanghai Clinical Center for Diabetes, Shanghai Sixth People's Hospital Affiliated to Shanghai Jiao Tong University School of Medicine, Shanghai 200233, China; Shanghai Diabetes Institute, Shanghai Key Laboratory of Diabetes Mellitus, Shanghai Clinical Center for Diabetes, Shanghai Sixth People's Hospital Affiliated to Shanghai Jiao Tong University School of Medicine, Shanghai 200233, China; Shanghai Diabetes Institute, Shanghai Key Laboratory of Diabetes Mellitus, Shanghai Clinical Center for Diabetes, Shanghai Sixth People's Hospital Affiliated to Shanghai Jiao Tong University School of Medicine, Shanghai 200233, China; Shanghai Diabetes Institute, Shanghai Key Laboratory of Diabetes Mellitus, Shanghai Clinical Center for Diabetes, Shanghai Sixth People's Hospital Affiliated to Shanghai Jiao Tong University School of Medicine, Shanghai 200233, China; Shanghai Diabetes Institute, Shanghai Key Laboratory of Diabetes Mellitus, Shanghai Clinical Center for Diabetes, Shanghai Sixth People's Hospital Affiliated to Shanghai Jiao Tong University School of Medicine, Shanghai 200233, China; Department of Endocrinology, Affiliated Hospital of Nantong University, Nantong 226001, China; Shanghai Diabetes Institute, Shanghai Key Laboratory of Diabetes Mellitus, Shanghai Clinical Center for Diabetes, Shanghai Sixth People's Hospital Affiliated to Shanghai Jiao Tong University School of Medicine, Shanghai 200233, China; Shanghai Diabetes Institute, Shanghai Key Laboratory of Diabetes Mellitus, Shanghai Clinical Center for Diabetes, Shanghai Sixth People's Hospital Affiliated to Shanghai Jiao Tong University School of Medicine, Shanghai 200233, China; Shanghai Diabetes Institute, Shanghai Key Laboratory of Diabetes Mellitus, Shanghai Clinical Center for Diabetes, Shanghai Sixth People's Hospital Affiliated to Shanghai Jiao Tong University School of Medicine, Shanghai 200233, China; Shanghai Diabetes Institute, Shanghai Key Laboratory of Diabetes Mellitus, Shanghai Clinical Center for Diabetes, Shanghai Sixth People's Hospital Affiliated to Shanghai Jiao Tong University School of Medicine, Shanghai 200233, China; Institute for Metabolic Disease, Fengxian Central Hospital Affiliated to Southern Medical University, Shanghai 201499, China

**Keywords:** molecular diagnosis, monogenic diabetes mellitus, monogenic obesity, targeted panel, whole-exome sequencing

## Abstract

A small fraction of patients diagnosed with obesity or diabetes mellitus has an underlying monogenic cause. Here, we constructed a targeted gene panel consisting of 83 genes reported to be causative for monogenic obesity or diabetes. We performed this panel in 481 patients to detect causative variants and compared these results with whole-exome sequencing (WES) data available for 146 of these patients. The coverage of targeted gene panel sequencing was significantly higher than that of WES. The diagnostic yield in patients sequenced by the panel was 32.9% with subsequent WES leading to three additional diagnoses with two novel genes. In total, 178 variants in 83 genes were detected in 146 patients by targeted sequencing. Three of the 178 variants were missed by WES, although the WES-only approach had a similar diagnostic yield. For the 335 samples only receiving targeted sequencing, the diagnostic yield was 32.2%. In conclusion, taking into account the lower costs, shorter turnaround time, and higher quality of data, targeted sequencing is a more effective screening method for monogenic obesity and diabetes compared to WES. Therefore, this approach could be routinely established and used as a first-tier test in clinical practice for specific patients.

## Introduction

Obesity has become a major public health issue in many countries, including China and increases the likelihood of various diseases such as type 2 diabetes mellitus (T2DM) that are associated with increased mortality (Swinburn et al., 2011). Although some obese individuals never develop T2DM and some individuals with T2DM are not obese (Tanamas et al., 2018), the increase in obesity prevalence has been linked with rising trends in T2DM prevalence (Mokdad et al., 2003). Patients with obesity or diabetes often share similar clinical characteristics such as insulin resistance, making it much more difficult to establish an aetiological diagnosis and corresponding treatment. These two metabolic disorders arise from the interplay between an at-risk genetic profile and environmental risk factors. A small proportion of obesity and diabetes can present as monogenic conditions, where genetic factors play a greater role with less dependency on environmental triggers.

Monogenic obesity is a type of rare and severe early-onset obesity, resulting from a single mutation of a gene such as *MC4R, LEP, LEPR*, and *POMC* primarily involved in the leptin–melanocortin pathway (van der Klaauw and Farooqi, 2015). Syndromic monogenetic forms of obesity often present with severe obesity and associated disorders such as developmental delay and learning difficulties. Over 100 monogenic obesity syndromes are known, one famous example being the Bardet–Biedl syndrome (BBS). Identifying these genetic mutations is important to plan personalized clinical management. For instance, patients with causative *LEP* gene mutations should receive dietary management and leptin replacement therapy (Styne et al., 2017), whereas those with *LEPR, PCSK1*, or *POMC* deficiency can take the drug setmelanotide, a selective *MC4R* agonist that was recently approved by the FDA (Yeo et al., 2021). A previous study in our laboratory showed that monogenic obesity mutation carriers had less weight loss over both short-term and long-term periods after bariatric surgery compared to non-carriers, highlighting the importance of genetic diagnosis for obesity before surgical intervention (Li et al., 2019).

Monogenic diabetes, accounting for ∼1%–5% of diabetes, is caused by a single mutation in one of >40 genes reported to date (Hattersley et al., 2018). The majority of monogenic diabetes categories are maturity-onset diabetes of the young (MODY), neonatal diabetes mellitus (NDM), and syndromic diabetes such as Wolfram syndrome and lipodystrophy. Misdiagnosis and subsequent non-indicated drug use often happen because of overlapping phenotypes with type 1 diabetes mellitus (T1DM), such as young onset and leanness, or T2DM, such as preserved β cell function and similar family history. The average time from diabetes diagnosis to obtaining a definitive genetic diagnosis in patients with MODY is ∼13 years, during which patients are treated as T1DM or T2DM but their glucose levels are not well controlled (Thanabalasingham and Owen, 2011). Therefore, correct molecular diagnosis plays a substantial role in proper diagnosis and subsequent treatment. For example, patients with HNF1A-MODY have an increased sensitivity to sulfonylureas rather than other hypoglycaemic drugs, whereas those with GCK-MODY improve glycaemic control by diet management and exercise without drug administration (Thanabalasingham and Owen, 2011).

Precision medicine, first put forward in 2008, suggests that clinicians should make a diagnosis based on molecular detection instead of their clinical experience (Katsnelson, 2013). Previously, a molecular diagnosis was usually performed through Sanger sequencing of one or several common candidate genes. However, the increasing number of genes implicated in monogenic obesity or diabetes renders this approach of sequencing single genes less useful. With the development of next-generation sequencing, whole-genome sequencing (WGS) and whole-exome sequencing (WES) have successfully identified numerous causative variants (McCombie et al., 2019). However, large-scale genetic screening has not yet become a standard part of the diagnostic work-up due to high costs, large data volumes, and complex data analyses. Since lower costs and simpler workflow are two major issues in clinical diagnosis both for patients and clinicians, new diagnostic tools are urgently needed. Targeted sequencing with panels of specific disease-causing genes rather than the whole genome or exome offers an alternative. In a few studies, researchers have performed targeted gene panels for patients diagnosed with suspected monogenetic obesity (Kleinendorst et al., 2018; Roberts et al., 2022) or diabetes (Park et al., 2019; Lee et al., 2021), especially MODY, and obtained considerable genetic diagnostic yields. However, none of the panels contained both genes causative for obesity and diabetes, and none of them was applied to Chinese patients. In addition, there is still a lack of research addressing the use of targeted panels versus WES; thus, clinicians are faced with the challenge of making decisions without sufficient information.

Here, we constructed a targeted gene panel with 83 genes causative for monogenic obesity or diabetes and applied this panel sequencing in 481 patients with suspected monogenic obesity or diabetes. We also performed WES in 146 of these patients. This study aimed to evaluate the applicability of targeted gene panel sequencing in the screening for monogenic obesity and diabetes, compare the quality of variant detection by targeted gene panel sequencing with that by WES, and help patients with causative mutations and potentially deleterious variants in diagnosis, treatment, and genetic counselling.

## Results

### Patient characteristics

A total of 481 patients received sequencing tests in this study, of which 146 were sequenced by both the targeted gene panel and WES. Based on both gene detection and clinical evaluation, 102 of 146 patients were finally diagnosed with obesity, of which 19 had accompanying diabetes mellitus. An additional group of 30 patients was diagnosed with MODY. These three conditions accounted for 90.4% of all cases in this study. The remaining patients were diagnosed with NDM or syndromic diabetes. The age of the patients ranged from 2 to 70 years, with a median of 28 years. Another 335 patients only received targeted gene panel sequencing. The characteristics of all patients are summarized in Supplementary Table S1.

### Coverage of targeted gene panel sequencing versus WES

**Figure 1:**
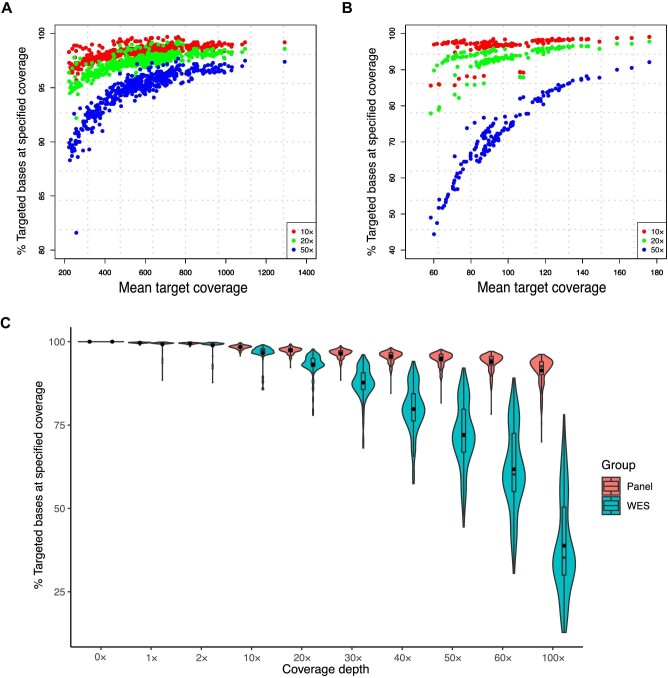
Coverage analysis for targeted gene panel sequencing and WES. (**A** and **B**) The *x*-axis indicates the mean target coverage for each sample subjected to panel sequencing (**A**) or WES (**B**), and the *y*-axis indicates the fraction of target base pairs that has at least 10×, 20×, and 50× coverage for each sample. (**C**) Sequencing coverage comparison between targeted gene panel sequencing and WES.

Sequencing depth and coverage analysis for each sample sequenced by the targeted panel or WES are shown in Figure 1. Samples of 481 patients were processed for targeted gene panel sequencing, achieving an average depth of 576.6×. An average of 98.39%, 97.38%, and 94.82% of targeted samples had at least 10×, 20×, and 50× coverage, respectively (Figure 1A). In comparison, 146 samples were subjected to WES, and the average depth of coverage for the whole-exome region was 96.7×. An average of 96.54%, 93.14%, and 72.05% of bases had at least 10×, 20×, and 50× coverage, respectively (Figure 1B). Overall, the samples subjected to targeted panel sequencing had better target region coverage than those subjected to WES, which is more obvious when comparing coverage with at least 50× (Figure 1C).

**Figure 2:**
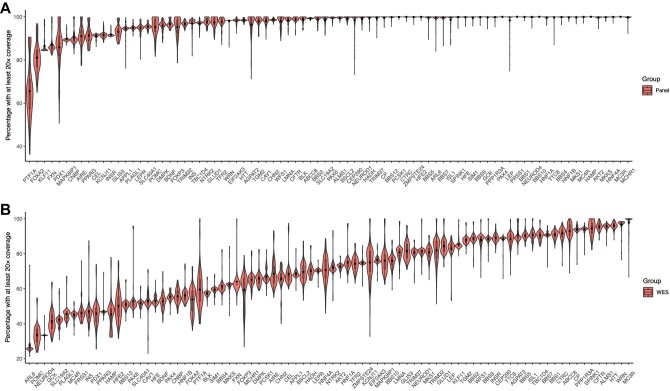
Overview of targeted genes with at least 20× coverage in targeted gene panel sequencing (**A**) and WES (**B**). Genes are ranked by the coverage percentage.

We next analysed the sequencing coverage of every single gene (Figure 2). Among the 83 target genes, 51 genes were >90% covered by at least 20× across all samples subjected to targeted gene panel sequencing, of which 4 genes (*MC3R, BBS10, HAMP*, and *PRSS1*) were 100% covered. The average percentages with at least 20× coverage in the panel were mostly >80%, except for the gene *PTF1A*, which had the lowest average percentage (65.6%). *PTF1A* had an even lower percentage (59.5%) in WES. However, only 16 genes were covered >90% with at least 20× coverage across all patients subjected to WES, 12 genes had a <50% average percentage, and 38 genes had a <80% average percentage. These results demonstrated that genes subjected to the targeted gene panel had significantly better coverage in general than those subjected to WES.

### Diagnostic yields of the targeted gene panel and WES

**Figure 3:**
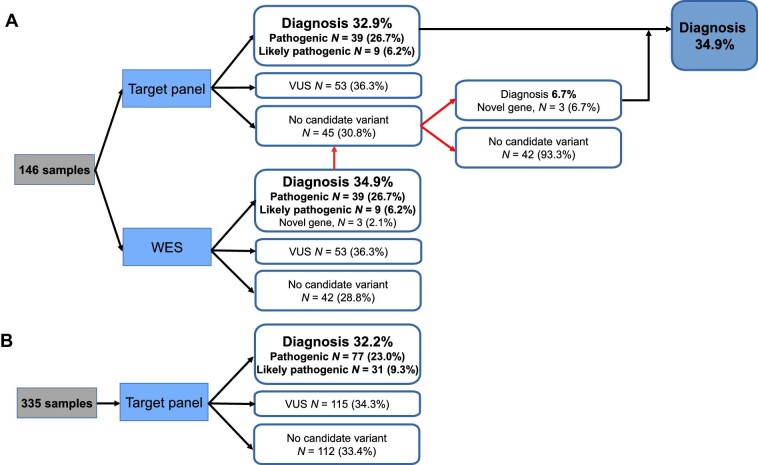
Diagnostic yield of targeted gene panel sequencing and WES. Positive cases refer to those identified pathogenic or likely pathogenic variants. Diagnostic yield was calculated through dividing the number of positive cases by that of total cases. (**A**) In total, 146 samples were sequenced by the targeted gene panel and subsequent WES. (**B**) A total of 335 samples only received targeted gene panel sequencing.

In the targeted panel, 39 pathogenic and 9 likely pathogenic variants were successfully identified in 48 out of 146 patients, achieving a total diagnostic rate of 32.9% (Figure 3A). Besides, variants of uncertain significance (VUSs) were identified in 53 patients. In 45 patients, no candidate variant was detected. Subsequent WES of these 45 unsolved cases identified three additional diagnoses with two novel genes in addition to the selected 83 genes, increasing the total diagnostic yield to 34.9% (51 out of 146 patients). For samples only receiving targeted gene panel sequencing, the diagnostic yield was 32.2% (Figure 3B), which confirmed the stability and consistency of the targeted sequencing approach. In comparison, the diagnostic yield of the WES-only approach was 34.9%. These data reflected that targeted gene panel sequencing and WES shared similar diagnostic yields in this study.

In addition, the number of variants for each sample after filtering was also comparable between panel and WES (Supplementary Figure S1). However, variants detected by the panel or WES were not exactly the same (Supplementary Table S2). Among the variants detected by the panel, three were missed by WES. For example, in a patient diagnosed with obesity, a likely pathogenic frameshift in *TBC1D4* was detected by targeted gene panel sequencing, yet WES failed to obtain this important genetic diagnosis. Another patient was identified a variant in MODY-related gene *HNF4A*, but WES missed this important identification either.

### Positive variants

**Figure 4:**
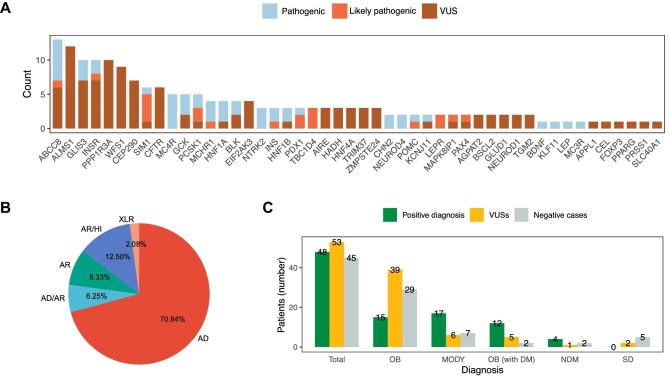
Detailed information on detected variants. (**A**) Genes are sorted by the number of detected variants. (**B**) Percentage of inheritance patterns for detected genes. (**C**) Distribution of patients with identified pathogenic and likely pathogenic variants (positive diagnosis), VUSs, and negative cases, classified by disease subtype. AD, autosomal dominant; AR, autosomal recessive; DM, diabetes mellitus; HI, haplo insufficient; OB, obesity; SD, syndromic diabetes; XLR, X-linked recessive.

Based on the targeted panel sequencing results, 178 variants of the 83 panel genes meeting the American College of Medical Genetics and Genomics (ACMG) criteria were conclusively identified in 146 patients (Figure 4A). The majority of diagnoses (70.84%) were autosomal dominant (Figure 4B). For patients diagnosed with obesity, the most common pathogenic variants were detected in the *MC4R* gene, which were identified in four patients. Among these, a 16-year-old patient diagnosed with severe obesity (body mass index [BMI] of 84.5 kg/m^2^) had two pathogenic variants in the same gene, namely p.G98R and p.C277X in the *MC4R* gene (Table 1). He was first diagnosed with obesity at the age of 1 year and became more and more obese since then. Notably, his sister with a BMI of 74.31 kg/m^2^ was diagnosed with MC4R-obesity as well. Pathogenic variants in the *MCHR1* gene were detected in three patients, followed by variants in *NTRK2, PCSK1*, and *CHN2*, which were each detected in two samples. Interestingly, two novel pathogenic variants (p.Y706S and p.Y706X) in the *NTRK2* gene were detected in one patient whose BMI was 42.1 kg/m^2^ with an onset age of 7 years. For each of the genes *LEP, MC3R, POMC, BDNF*, and *SIM1*, only one pathogenic variant was identified, whereas none of the patients harboured a pathogenic *LEPR* variant.

**Table 1:** Detected variants of 146 patients by targeted gene panel.

**Patient no.**	**Age (years)**	**Sex^a^**	**BMI (kg/m^2^)**	**Initial diagnosis**	**Age of onset (years)**	**Final diagnosis**	**Final variant classification**	**Variant classification**	**Gene**	**Position**	**SNP**	**Ref/Alt**	**GERP**	**AA change**	**Pathogenic prediction^b^**	**MAF in EAS^c^**
1	16	1	36.1	OB (with DM)	.(16)	MODY	P	P	*ABCC8*	11:17449895	rs750182645	C/T	3.22	NM_000352:exon14:c.G1981A:p.G661S	D	0
2	33	1	42.0	OB	26		VUS	VUS	*ALMS1*	2:73682365	.	A/C	4.19	NM_015120:exon9:c.A7614C:p.K2538N	D	Novel
								VUS	*ALMS1*	2:73826532	.	C/A	−1.92	NM_015120:exon17:c.C11549A:p.A3850E	T	Novel
								VUS	*INSR*	19:7184420	rs201147780	T/C	3.12	NM_000208:exon3:c.A881G:p.K294R	D	0.15%
4	19	1	53.8	OB	11		VUS	VUS	*INSR*	19:7125355	rs372010924	C/T	5.06	NM_001079817:exon16:c.G3161A:p.R1054Q	DVS	0
5	25	1	46.6	OB	5		VUS	VUS	*PAX4*	7:127253578	rs114315130	G/A	4.24	NM_006193:exon5:c.C547T:p.R183C	DS	0.56%
6	29	2	35.8	OB (with DM)	.(26)		P	P	*MCHR1*	22:41077454	rs777859258	G/A	5.24	NM_005297:exon2:c.G791A:p.G264D	D	0.03%
								VUS	*PPP1R3A*	7:113558701	.	T/G	4.88	NM_002711:exon1:c.A351C:p.E117D	D	Novel
								VUS	*PPP1R3A*	7:113519261	rs200678247	T/A	2.14	NM_002711:exon4:c.A1886T:p.D629V	D	0.6%
								LP	*SIM1*	6:100838745	rs199641634	G/C	4.95	NM_005068:exon11:c.C1793G:p.A598G	D	0.38%
7	19	2	35.6	OB	7		VUS	VUS	*GLIS3*	9:3856011	rs140981222	T/C	5.8	NM_152629:exon8:c.A2006G:p.H669R	D	0.71%
8	50	2	50.8	OB (with DM)	35(49)		VUS	VUS	*ZMPSTE24*	1:40747152	rs775139554	C/T	4.74	NM_005857:exon7:c.C907T:p.R303C	D	0
10	27	2	39.9	OB	11		P	P	*NTRK2*	9:87338488	rs764986674	G/T	4.7	NM_001018064:exon6:c.G584T:p.G195V	Splicing	0.01%
11	28	2	34.8	OB	7		P	P	*LEP*	7:127894640	rs1800564	G/A	2.49	NM_000230:exon3:c.G328A:p.V110M	DW	0.02%
12	30	1	53.5	OB (with DM)	.(29)		VUS	VUS	*TGM2*	20:36760744	rs201666703	G/A	2.3	NM_001323317:exon10:c.C1531T:p.R511W	DS	0.44%
14	27	2	39.5	OB (with DM)	17(25)		LP	LP	*INS*	11:2182135	rs13306444	C/T	−0.256	NM_001185098:exon1:c.G67A:p.A23T	D	0.6%
								VUS	*CFTR*	7:117251784	rs201591901	C/T	5.69	NM_000492:exon20:c.C3289T:p.R1097C	DS	0.24%
15	21	1	46.8	OB	5		P	P	*PCSK1*	5:95735706	rs780394907	C/T	5.48	NM_000439:exon10:c.G1381A:p.V461M	DS	0
16	31	1	42.7	OB (with DM)	13(28)		P	P	*GLIS3*	9:3829448	rs201704428	C/T	5.92	NM_152629:exon9:c.G2053A:p.D685N	D	0.02%
								LP	*MCHR1*	22:41077832	rs190547628	G/A	5.4	NM_005297:exon2:c.G1169A:p.R390H	D	0.74%
								LP	*PDX1*	13:28494372	rs192902098	C/G	5.47	NM_000209:exon1:c.C97G:p.P33A	DS	0.41%
17	27	2	35.1	OB	2		P	P	*MCHR1*	22:41077825	rs774688778	C/T	5.4	NM_005297:exon2:c.C1162T:p.R388C	D	0.01%
								VUS	*ALMS1*	2:73717455	.	A/C	3.48	NM_015120:exon10:c.A8366C:p.E2789A	D	Novel
								VUS	*ALMS1*	2:73747095	rs200432874	G/A	4.75	NM_015120:exon11:c.G9730A:p.A3244T	D	0.16%
19	27	2	52.9	OB (with DM)	6(26)	MODY	P	P	*HNF1A*	12:121416752	.	C/T	4.34	NM_000545:exon1:c.C181T:p.P61S	DS	Novel
								P	*NEUROD4*	12:55420720	rs372637889	C/T	5.56	NM_021191:exon2:c.C497T:p.T166I	DS	0.01%
								LP	*MAPK8IP1*	11:45926356	rs549861347	G/A	4.87	NM_005456:exon9:c.G1864A:p.V622I	D	0.15%
20	28	2	42.8	OB (with DM)	7(25)		LP	LP	*SIM1*	6:100868686	rs188821440	T/C	5.81	NM_005068:exon9:c.A1147G:p.R383G	DS	0.17%
								LP	*SIM1*	6:100841606	rs755621081	T/C	−2.1	NM_005068:exon10:c.A1327G:p.S443G	T	0.03%
21	30	1	52.0	OB	23		VUS	VUS	*NEUROD1*	2:182542816	rs751374620	C/T	6.02	NM_002500:exon2:c.G772A:p.E258K	D	0.02%
24	50	1	36.8	MODY	35		P	P	*NEUROD4*	12:55420465	rs764006660	G/A	5.37	NM_021191:exon2:c.G242A:p.R81H	DVS	0
								LP	*LEPR*	1:66075945	rs777166726	T/C	−0.69	NM_001198687:exon13:c.T1961C:p.M654T	T	0.01%
27	.	2	.	MODY	.		VUS	VUS	*SIM1*	6:100901687	.	G/A	5.56	NM_005068:exon2:c.C209T:p.T70I	D	Novel
								VUS	*CEP290*	12:88472979	rs748471942	G/A	2.26	NM_025114:exon39:c.C5254T:p.R1752W	DS	0
								VUS	*CEP290*	12:88522737	.	C/G	5.2	NM_025114:exon11:c.G928C:p.V310L	DS	Novel
28	.	2	.	NDM	.		VUS	VUS	*AIRE*	21:45708284	rs74162061	G/A	0.359	NM_000383:exon5:c.G595A:p.V199I	DW	0.15%
30	31	2	42.6	MODY	31		VUS	VUS	*WFS1*	4:6303542	rs200672755	G/A	5.49	NM_001145853:exon8:c.G2020A:p.G674R	DS	0.02%
								VUS	*EIF2AK3*	2:88874416	rs200955126	G/A	5.56	NM_001313915:exon13:c.C2132T:p.T711I	D	0.09%
31	19	1	23.5	MODY	17		VUS	VUS	*EIF2AK3*	2:88892887	rs753157985	A/G	5.86	NM_001313915:exon4:c.T217C:p.W73R	D	0.05%
32	18	1	24.7	MODY	17		VUS	VUS	*PCSK1*	5:95759020	.	G/T	1.84	NM_000439:exon4:c.C540A:p.N180K	DS	Novel
								VUS	*ALMS1*	2:73613245	rs772450464	C/A	3.69	NM_015120:exon1:c.C249A:p.H83Q	D	0.35%
								VUS	*ALMS1*	2:73677903	rs199615803	C/T	2.17	NM_015120:exon8:c.C4246T:p.R1416W	D	0.19%
34	18	1	26.0	MODY	16		LP	LP	*PDX1*	13:28494372	rs192902098	C/G	5.47	NM_000209:exon1:c.C97G:p.P33A	DS	0.41%
								LP	*ABCC8*	11:17450014	.	T/C	.	NM_001351295:exon14:c.A1928G:p.E643G	.	0.06%^d^
35	11	1	25.8	NDM	11		P	P	*ABCC8*	11:17482154	rs144705160	G/A	4.54	NM_000352:exon6:c.C892T:p.R298C	DS	0.03%
36	17	1	43.2	MODY	17		VUS	VUS	*PPP1R3A*	7:113517860	rs771586895	A/G	3.49	NM_002711:exon4:c.T3287C:p.I1096T	D	0.07%
40	23	2	22.1	MODY	13		P	P	*INS*	11:2182047	.	G/A	3.08	NM_001185098:exon1:c.C155T:p.P52L	DS	Novel
41	18	1	.	MODY	.		P	P	*BLK*	8:11421519	.	C/T	2.85	NM_001330465:exon12:c.C1207T:p.R403C	DVS	Novel
42	20	1	48.7	MODY	20		P	P	*ABCC8*	11:17449416	.	C/T	1.82	NM_000352:exon15:c.G2114A:p.R705Q	D	0^d^
								P	*PCSK1*	5:95761592	.	GT/G	.	NM_000439:exon3:c.327delA:p.K109Nfs*5	Frameshift	Novel
								P	*CHN2*	7:29539513	.	C/T	5.61	NM_001039936:exon3:c.C362T:p.S121F	DS	Novel
43	14	2	17.3	MODY	14		P	P	*PDX1*	13:28498720	.	G/A	3.31	NM_000209:exon2:c.G734A:p.G245E	D	Novel
								VUS	*HADH*	4:108911187	rs74428123	C/G	1.91	NM_001184705:exon1:c.C99G:p.I33M	DS	0.55%
45	23	1	24.3	MODY	23		P	P	*ABCC8*	11:17418784	rs766624549	T/C	5.07	NM_000352:exon32:c.A3944G:p.H1315R	D	0
								P	*HNF1A*	12:121431427	.	C/T	4.55	NM_000545:exon3:c.C631T:p.Q211X	Stopgain	Novel
46	.	1	.	MODY	.		VUS	VUS	*TRIM37*	17:57076784	rs774360780	C/T	4.11	NM_001320987:exon23:c.G2747A:p.G916D	DW	0.02%
47	25	1	19.0	MODY	24		P	P	*GCK*	7:44185238	.	AGGCGC/A	.	NM_000162:exon9:c.1106_1110del:p.R369Lfs*88	Frameshift	Novel
								VUS	*EIF2AK3*	2:88882979	.	T/G	1.85	NM_001313915:exon10:c.A1279C:p.N427H	D	Novel
48	34	2	26.2	MODY	.		P	P	*HNF1B*	17:36091805	rs121918672	G/A	3.55	NM_000458:exon4:c.C826T:p.R276X	Stopgain	Novel
49	.	1	.	NDM	.		P	P	*ABCC8*	11:17426072	rs797045209	G/A	5.32	NM_000352:exon28:c.C3544T:p.R1182W	DVS	Novel
								VUS	*PPP1R3A*	7:113558894	.	T/A	3.81	NM_002711:exon1:c.A158T:p.Y53F	D	Novel
50	.	1	.	NDM	.		P	P	*INS*	11:2182098	.	A/T	2.67	NM_001185098:exon1:c.T104A:p.L35Q	DS	Novel
51	2	2	.	NDM	2		P	P	*GCK*	7:44187276	.	T/G	5.5	NM_000162:exon7:c.A836C:p.E279A	D	Novel
52	17	1	46.0	OB	7		VUS	VUS	*GLIS3*	9:3828294	.	G/A	5.92	NM_152629:exon10:c.C2306T:p.S769F	D	Novel
54	29	2	45.0	OB	9		VUS	VUS	*ABCC8*	11:17449470	rs150316347	G/A	4.33	NM_000352:exon15:c.C2060T:p.T687M	DS	0.02%
55	37	1	41.0	OB (with DM)	30(.)	MODY	P	P	*GCK*	7:44186084	.	G/A	4.77	NM_000162:exon8:c.C997T:p.R333C	DS	Novel
56	23	2	42.2	OB	13		VUS	VUS	*ABCC8*	11:17414592	.	C/A	0.891	NM_000352:exon39:c.G4692T:p.K1564N	D	Novel
57	26	1	37.7	OB	18		VUS	VUS	*TRIM37*	17:57093160	rs566626073	C/G	2.87	NM_001320987:exon20:c.G2285C:p.G762A	D	0.15%
								VUS	*AGPAT2*	9:139568296	rs1003229436	G/A	−1.08	NM_001012727:exon5:c.C649T:p.H217Y	D	Novel
59	34	2	37.1	OB	24		VUS	VUS	*AIRE*	21:45706935	.	C/A	3.21	NM_000383:exon3:c.C382A:p.L128I	D	Novel
								VUS	*HADH*	4:108944651	rs750069218	A/G	5.76	NM_001184705:exon5:c.A568G:p.S190G	DS	0.01%
61	31	2	37.7	OB	28		VUS	VUS	*HNF1B*	17:36104659	.	G/A	3.1	NM_000458:exon1:c.C217T:p.R73C	DS	Novel
								VUS	*GLIS3*	9:4118540	rs35154632	C/G	5.59	NM_152629:exon3:c.G473C:p.G158A	D	0.24%
62	30	2	46.0	OB	14		P	P	*BDNF*	11:27722541	rs753114863	G/A	.	NM_001143809:exon1:c.C43T:p.R15C	.	0
63	25	1	38.2	OB	5		LP	LP	*TBC1D4*	13:75900450	.	C/T	5.48	NM_001286658:exon10:c.G1916A:p.R639Q	D	Novel
64	33	2	34.8	OB	27		VUS	VUS	*PPP1R3A*	7:113558284	.	T/A	2.13	NM_002711:exon1:c.A768T:p.L256F	D	Novel
65	27	2	46.6	OB	7		P	P	*MC4R*	18:58038752	rs769342968	A/T	1.01	NM_005912:exon1:c.T831A:p.C277X	Stopgain	0.01%
								VUS	*INSR*	19:7184420	rs201147780	T/C	3.12	NM_000208:exon3:c.A881G:p.K294R	D	0.15%
								VUS	*NEUROD1*	2:182543532	.	G/C	5.37	NM_002500:exon2:c.C56G:p.P19R	D	Novel
66	20	2	39.9	OB (with DM)	18(.)	MODY	P	P	*BLK*	8:11400837	rs765828168	C/A	5.54	NM_001715:exon2:c.C104A:p.A35D	DW	0.01%
								VUS	*PPP1R3A*	7:113522130	rs756879447	T/A	−1.41	NM_002711:exon3:c.A930T:p.E310D	D	0.01%
69	29	2	40.0	OB (with DM)	14(.)		P	P	*INSR*	19:7142863	.	C/T	4.24	NM_001079817:exon11:c.G2470A:p.V824M	D	Novel
70	26	2	41.0	OB	6		VUS	VUS	*ALMS1*	2:73678023	rs373453289	G/T	−2.36	NM_015120:exon8:c.G4366T:p.V1456F	D	0
								VUS	*ALMS1*	2:73717281	rs200859630	T/C	2.98	NM_015120:exon10:c.T8192C:p.V2731A	D	0.05%
71	26	1	37.2	OB (with DM)	6(25)		VUS	VUS	*GLUD1*	10:88854144	.	G/C	4.25	NM_005271:exon1:c.C383G:p.S128C	D	Novel
72	.	1	.	OB	.		VUS	VUS	*GLIS3*	9:3829360	.	G/T	5.93	NM_152629:exon9:c.C2141A:p.A714D	D	Novel
								VUS	*GLIS3*	9:3898768	rs542599450	G/C	4.82	NM_152629:exon6:c.C1586G:p.P529R	D	0.01%
73	.	1	.	OB	.		P	P	*MC3R*	20:54824533	rs61735256	C/T	4.91	NM_019888:exon1:c.C634T:p.L212F	D	0.08%
								LP	*TBC1D4*	13:75898423	.	GC/G	.	NM_014832:exon11:c.2147delG:p.S716Tfs*43	Frameshift	0
75	35	2	40.1	OB	15		VUS	VUS	*BSCL2*	11:62458766	rs773431994	G/A	3.38	NM_001122955:exon7:c.C991T:p.R331C	DS	0
								VUS	*BLK*	8:11412981	rs200515443	G/A	4.49	NM_001330465:exon7:c.G547A:p.E183K	DS	0.02%
								VUS	*HNF4A*	20:43019284	rs780566668	C/T	4.15	NM_001287182:exon2:c.C5T:p.S2L	D	0
76	25	1	47.3	OB	5		VUS	VUS	*CEP290*	12:88483024	rs62640581	G/A	2.64	NM_025114:exon31:c.C3814T:p.R1272X	Stopgain	Novel
								VUS	*CEP290*	12:88514827	rs551533386	C/T	5.84	NM_025114:exon14:c.G1306A:p.E436K	D	0.06%
								VUS	*CFTR*	7:117251784	rs201591901	C/T	5.69	NM_000492:exon20:c.C3289T:p.R1097C	DS	0.24%
79	21	2	36.6	OB	1		VUS	VUS	*MAPK8IP1*	11:45924267	rs200600045	G/A	0.613	NM_005456:exon5:c.G949A:p.A317T	T	0
81	26	2	39.5	OB	1		VUS	VUS	*PRSS1*	7:142460731	.	G/A	3.32	NM_002769:exon5:c.G604A:p.G202S	D	Novel
								VUS	*GLUD1*	10:88854520	.	G/A	1.88	NM_005271:exon1:c.C7T:p.R3C	D	Novel
								VUS	*HNF4A*	20:43052672	rs201777208	A/G	5.4	NM_000457:exon8:c.A907G:p.S303G	D	0.08%
82	28	2	31.1	OB	8		VUS	VUS	*WFS1*	4:6304167	rs775303221	T/G	4.68	NM_001145853:exon8:c.T2645G:p.F882C	DS	0.02%
84	28	1	37.0	OB	12		VUS	VUS	*WFS1*	4:6296857	rs752585187	G/A	4.35	NM_001145853:exon7:c.G802A:p.D268N	D	0^d^
86	23	1	35.9	OB (with DM)	13(19)	MODY	P	P	*HNF1A*	12:121434170	rs757068809	C/T	5.06	NM_000545:exon5:c.C1061T:p.T354M	DS	0
								LP	*PAX4*	7:127251150	rs143084654	G/A	1.59	NM_006193:exon9:c.C1000T:p.P334S	D	0.17%
87	70	2	33.3	OB (with DM)	60(.)		VUS	VUS	*CEP290*	12:88472996	rs61941020	C/T	4.28	NM_025114:exon39:c.G5237A:p.R1746Q	D	0.7%
								VUS	*CEP290*	12:88520194	rs536127246	C/T	5.26	NM_025114:exon12:c.G964A:p.D322N	D	0.04%
								VUS	*CEP290*	12:88522780	.	T/G	1.14	NM_025114:exon11:c.A885C:p.K295N	D	Novel
								VUS	*KCNJ11*	11:17408977	rs768909861	C/A	5.29	NM_000525:exon1:c.G662T:p.R221L	.	Novel
89	41	2	45.7	OB	20		VUS	VUS	*ABCC8*	11:17449470	rs150316347	G/A	4.33	NM_000352:exon15:c.C2060T:p.T687M	DS	0.02%
90	30	1	46.1	OB (with DM)	15(.)		P	P	*INSR*	19:7141704	rs187282966	C/T	5.62	NM_001079817:exon12:c.G2630A:p.R877Q	D	0
91	51	1	34.5	OB	.		LP	LP	*SIM1*	6:100896091	.	T/C	4.39	NM_005068:exon7:c.A781G:p.I261V	T	Novel
					.			VUS	*ALMS1*	2:73675562	.	A/T	1.3	NM_015120:exon8:c.A1905T:p.L635F	D	Novel
								VUS	*ALMS1*	2:73799955	rs748200460	C/T	5.48	NM_015120:exon16:c.C10948T:p.H3650Y	D	0.01%
								VUS	*WFS1*	4:6296873	rs546948362	A/C	4.2	NM_001145853:exon7:c.A818C:p.E273A	D	0.09%
								VUS	*WFS1*	4:6303479	rs201064551	C/T	5.41	NM_001145853:exon8:c.C1957T:p.R653C	D	0.1%
92	37	1	39.5	OB	27		VUS	VUS	*CFTR*	7:117149143	rs115545701	C/T	4.77	NM_000492:exon3:c.C220T:p.R74W	D	0.1%
								VUS	*CFTR*	7:117235056	rs397508397	G/A	−7.78	NM_000492:exon15:c.G2563A:p.V855I	D	0.1%
93	30	1	34.3	OB	20		VUS	VUS	*ALMS1*	2:73718496	rs775701628	C/G	−1.52	NM_015120:exon10:c.C9407G:p.T3136S	T	0.01%
								VUS	*ALMS1*	2:73800390	.	C/T	4.79	NM_015120:exon16:c.C11383T:p.H3795Y	D	Novel
								VUS	*HNF4A*	20:43053014	rs145360792	G/A	0.802	NM_001030004:exon8:c.G1183A:p.A395T	D	0.11%
96	20	2	40.3	OB	12		VUS	VUS	*GLIS3*	9:4118346	.	C/A	2.17	NM_152629:exon3:c.G667T:p.G223C	DW	Novel
								VUS	*ZMPSTE24*	1:40737662	rs772647434	G/A	5.3	NM_005857:exon6:c.G724A:p.A242T	D	0.07%
97	30	1	41.0	OB	20		VUS	VUS	*HNF1A*	12:121437368	rs752219487	G/A	1.69	NM_000545:exon9:c.G1706A:p.S569N	D	0
98	33	1	62.8	OB	13		VUS	VUS	*INSR*	19:7142969	.	C/A	−5.21	NM_001079817:exon11:c.G2364T:p.K788N	D	Novel
								VUS	*INSR*	19:7143036	.	G/C	2.17	NM_001079817:exon11:c.C2297G:p.A766G	T	Novel
								VUS	*ABCC8*	11:17482154	rs144705160	G/A	4.54	NM_000352:exon6:c.C892T:p.R298C	DS	0.03%
								VUS	*APPL1*	3:57302458	rs183787750	A/G	1.5	NM_012096:exon21:c.A1926G:p.I642M	T	0.39%
101	38	2	35.0	OB	28		VUS	VUS	*GCK*	7:44187396	rs764146649	T/C	4.18	NM_000162:exon7:c.A716G:p.Q239R	D	0.05%
								VUS	*ZMPSTE24*	1:40758225	rs116771294	C/T	5.22	NM_005857:exon10:c.C1312T:p.L438F	DS	0.12%
103	52	2	37.7	OB	32		VUS	VUS	*CEL*	9:135946532	rs529664291	C/T	−0.744	NM_001807:exon11:c.C1652T:p.A551V	D	0.09%
104	35	1	34.8	OB	34		LP	LP	*POMC*	2:25384089	.	C/T	−1.97	NM_000939:exon3:c.G665A:p.R222K	T	Novel
105	41	2	34.1	OB (with DM)	31(28)	MODY	P	P	*KLF11*	2:10188257	rs773720978	G/A	5.07	NM_001177716:exon3:c.G742A:p.E248K	D	0.03%
								LP	*LEPR*	1:66036167	rs754188832	G/C	0.867	NM_001198687:exon3:c.G52C:p.V18L	T	0.03%
106	37	1	40.9	OB (with DM)	35(.)	MODY	P	P	*HNF1B*	17:36093604	rs529294719	C/T	4.94	NM_000458:exon3:c.G755A:p.R252Q	D	0.01%
								VUS	*CFTR*	7:117251850	.	A/G	5.38	NM_000492:exon20:c.A3355G:p.I1119V	D	Novel
108	26	1	43.7	OB	16		P	P	*SIM1*	6:100838499	rs149142065	G/A	6.16	NM_005068:exon11:c.C2039T:p.S680L	D	0
								VUS	*INSR*	19:7117259	rs199599404	C/T	3.96	NM_001079817:exon21:c.G3921A:p.M1307I	D	0.01%
109	69	2	33.0	OB	.		VUS	VUS	*PPP1R3A*	7:113519261	rs200678247	T/A	2.14	NM_002711:exon4:c.A1886T:p.D629V	D	0.06%
								VUS	*TGM2*	20:36760809	rs149480979	G/A	2.66	NM_001323317:exon10:c.C1466T:p.P489L	D	Novel
110	27	2	41.0	OB	25		VUS	VUS	*CFTR*	7:117304869	rs397508670	C/T	5.87	NM_000492:exon25:c.C4091T:p.A1364V	DS	0
111	23	1	32.6	OB	.		P	P	*MC4R*	18:58038643	.	T/C	6.06	MC4R:NM_005912:exon1:c.A940G:p.K314E	D	Novel
								P	*MCHR1*	22:41077283	rs188147970	C/T	5.02	NM_005297:exon2:c.C620T:p.A207V	D	0.06%
								VUS	*ABCC8*	11:17432121	.	T/A	6.17	NM_000352:exon22:c.A2636T:p.D879V	DS	Novel
								VUS	*AIRE*	21:45712898	rs79212994	C/T	2.86	NM_000383:exon10:c.C1118T:p.A373V	D	0.06%
112	56	2	29.6	OB (with DM)	46(46)		LP	LP	*PCSK1*	5:95751742	rs183045011	A/G	5.92	NM_000439:exon6:c.T704C:p.V235A	DS	0.22%
								VUS	*PPP1R3A*	7:113518134	rs765187128	CCA/C	.	NM_002711:exon4:c.3011_3012del:p.V1004Gfs*15	Frameshift	0.02%
								VUS	*AGPAT2*	9:139569244	rs372408400	C/T	4.36	NM_001012727:exon4:c.G508A:p.V170M	DS	0
113	34	2	47.7	OB (with DM)	14(.)		P	P	*GLIS3*	9:4286245	.	T/C	5.75	NM_001042413:exon2:c.A181G:p.K61E	D	Novel
								VUS	*PPARG*	3:12421346	.	C/G	4.93	NM_001330615:exon2:c.C142G:p.P48A	T	Novel
114	49	2	32.1	OB (with DM)	29(.)		P	P	*POMC*	2:25387619	rs146551109	C/T	3.82	NM_000939:exon2:c.G23A:p.R8H	D	0
116	44	2	29.4	OB	40		VUS	VUS	*ABCC8*	11:17432067	rs563178863	T/A	6.17	NM_000352:exon22:c.A2690T:p.D897V	DS	0.1%^e^
								VUS	*BLK*	8:11407706	.	G/C	5.4	NM_001330465:exon5:c.G194C:p.R65T	DS	Novel
117	33	2	35.3	OB	23		VUS	VUS	*WFS1*	4:6290852	rs528811205	A/G	2.93	NM_001145853:exon4:c.A454G:p.R152G	D	0
118	50	2	28.5	OB (with DM)	48(48)		VUS	VUS	*EIF2AK3*	2:88890425	rs769083965	T/C	−0.046	NM_001313915:exon5:c.A460G:p.I154V	T	0
123	31	2	47.3	OB	21		VUS	VUS	*WFS1*	4:6279373	.	A/C	−5.04	NM_001145853:exon2:c.A191C:p.Q64P	D	Novel
124	28	2	29.3	OB	27		VUS	VUS	*GCK*	7:44191932	rs762922697	C/T	2.77	NM_000162:exon3:c.G301A:p.V101M	D	0
								VUS	*PPP1R3A*	7:113519261	rs200678247	T/A	2.14	NM_002711:exon4:c.A1886T:p.D629V	D	0.06%
								VUS	*GLIS3*	9:3829429	rs199505727	G/A	5	NM_152629:exon9:c.C2072T:p.P691L	D	0.44%
126	56	1	31.7	OB (with DM)	36(51)		LP	LP	*PCSK1*	5:95728863	rs140899352	C/T	1.88	NM_000439:exon14:c.G2104A:p.E702K	D	0.12%
								VUS	*SLC40A1*	2:190436515	rs987371643	C/G	5.29	NM_014585:exon5:c.G440C:p.S147T	D	Novel
127	34	2	40.2	OB (with DM)	29(.)		P	P	*CHN2*	7:29186538	.	A/G	−0.628	NM_001293069:exon2:c.A236G:p.H79R	D	Novel
								P	*GLIS3*	9:4118879	.	G/C	5.87	NM_152629:exon3:c.C134G:p.S45C	D	Novel
								LP	*INSR*	19:7125409	rs200921389	C/T	5.06	NM_001079817:exon16:c.G3107A:p.G1036D	D	0.1%
128	54	2	30.9	OB (with DM)	26(.)		P	P	*MC4R*	18:58039089	rs747681609	C/T	5.85	NM_005912:exon1:c.G494A:p.R165Q	DS	0
								P	*ABCC8*	11:17474773	rs771392416	C/T	5.79	NM_000352:exon7:c.G1069A:p.V357I	D	0
129	48	1	52.5	OB	33		LP	LP	*TBC1D4*	13:76055573	.	G/A	4.16	NM_001286658:exon1:c.C331T:p.Q111X	Stopgain	Novel
132	27	2	39.1	OB	9		VUS	VUS	*INSR*	19:7142938	rs35045353	C/T	5.56	NM_001079817:exon11:c.G2395A:p.G799S	D	0
133	64	2	45.4	OB	62		VUS	VUS	*TRIM37*	17:57138469	.	C/G	5.7	NM_001320987:exon11:c.G841C:p.D281H	Splicing	Novel
134	26	1	50.4	OB	12		VUS	VUS	*PPP1R3A*	7:113558921	rs201521408	C/T	6.17	NM_002711:exon1:c.G131A:p.R44Q	DS	0.06%
135	36	2	42.6	OB	11		VUS	VUS	*HADH*	4:108935686	rs377615662	G/A	5.84	NM_001184705:exon3:c.G361A:p.V121M	DS	0
136	.	1	.	OB	.		VUS	VUS	*FOXP3*	X:49110419	rs782731811	C/T	5.46	NM_001114377:exon8:c.G821A:p.R274Q	DS	0.03%
137	16	1	84.5	OB	1		P	P	*MC4R*	18:58039291	rs2282556	C/T	5.86	NM_005912:exon1:c.G292A:p.G98R	D	0
								P	*MC4R*	18:58038752	rs769342968	A/T	1.01	NM_005912:exon1:c.T831A:p.C277X	Stopgain	0.01%
140	55	1	41.1	OB (with DM)	.(35)	MODY	P	P	*KCNJ11*	11:17408580	.	G/T	3.35	NM_000525:exon1:c.C1059A:p.H353Q	D	Novel
141	35	1	24.2	SD	.		VUS	VUS	*WFS1*	4:6302559	rs773900146	C/T	4.84	NM_001145853:exon8:c.C1037T:p.P346L	D	0.01%
								VUS	*WFS1*	4:6302831	rs147974629	G/A	−5.5	NM_001145853:exon8:c.G1309A:p.G437S	D	0.8%
142	4	2	27.5	SD	.		VUS	VUS	*BSCL2*	11:62469968	.	A/G	5.64	NM_001122955:exon3:c.T458C:p.V153A	D	Novel
145	47	1	42.1	OB	7		P	P	*NTRK2*	9:87570425	.	A/C	4.98	NM_001018064:exon15:c.A2117C:p.Y706S	DS	Novel
								P	*NTRK2*	9:87570426	.	C/A	4.98	NM_001018064:exon15:c.C2118A:p.Y706X	Stopgain	Novel

SNP, single-nucleotide polymorphism; Ref, reference allele; Alt, alternative/variant allele; OB, obesity; DM, diabetes mellitus; SD, syndromic diabetes; P, pathogenic; LP, likely pathogenic.

a 1 represents male, 2 represents female.

b Pathogenic prediction was assessed according to CADD, DANN, MetaSVM, Polyphen2, SIFT, and M-CAP comprehensively. DVS, damaging very strongly; DS, damaging strongly; D, damaging; DW, damaging weakly; T, tolerated.

c MAF in East Asian population was referred to in the ExAC database. ‘Novel’ means the variant not found in any database among oneKG, ExAC, gnomAD, and esp6500.

d MAF was not shown in the ExAC database but in the gnomAD database.

e MAF was not shown in the ExAC database but in the oneKG database.

Among all variants, the *ABCC8* gene had the largest number of pathogenic variants with six variants in six different patients; thus, these patients were given a genetic diagnosis for MODY12. Other common pathogenic variants for MODY were identified in the genes *GCK* and *HNF1A*, variants of which were identified in three different patients each. Besides, two pathogenic variants were detected in each of the genes *HNF1B, INS, NEUROD4*, and *BLK*. Only one patient each harboured a single pathogenic variant in the gene *KCNJ11, PDX1, or KLF11.* Interestingly, some patients with MODY had more than one pathogenic MODY variant, such as patients no. 19 and no. 45 (Table 1). In total, 24 pathogenic variants were identified. Among these, five were novel in East Asians and 14 were novel in all populations. They have, to the best of our knowledge, not been reported in the published literature and databases.

Notably, some patients carried both obesity- and MODY-associated gene variants. One good example is patient no. 42 (Table 1) who had pathogenic variants of the MODY-related gene *ABCC8* and the obesity-associated genes *PCSK1* and *CHN2*.

In addition to the 83 panel genes, WES identified three variants of two novel genes in three samples from obese participants, namely *PRDM16* and *KSR2*, which were interpreted as pathogenic or likely pathogenic (Supplementary Table S3).

### Variants of unknown significance

About two-thirds of the variants were termed VUS in our study. VUSs were considered likely to play a role in disease, but their exact functional impact remained uncertain due to not fitting the expected inheritance mode, a relatively high minor allele frequency (MAF) in East Asian populations, or not being consistent with clinical information. A large number of the patients with obesity were carriers of VUSs in one or more of those obesity-related genes, among which BBS-related variants were an important part. In addition, some patients carrying variants of MODY-related genes did not show any blood glucose abnormalities but were only obese, hence these variants were classified as VUS as well.

*ALMS1*, which has been reported to cause Alström syndrome, had 12 variants in our study (Figure 4C). However, some patients carrying these variants did not show characteristic Alström syndromic features such as hearing loss and visual disturbance, and we failed to perform sequencing for their parents as this disease is autosomal recessive. Therefore, variants in *ALMS1* were all classified as VUS.

Besides, no variant was identified in 45 patients, more than half of which were diagnosed with obesity. Most of the patients with suspected syndromic diabetes had no detectable variant either.

## Discussion

In this study, we performed targeted panel sequencing of 83 genes in 481 patients with obesity or diabetes and compared the results of a subset of 146 patients with the corresponding WES data regarding target coverage and detected variants. To the best of our knowledge, this is the first direct comparison of targeted gene panel sequencing and WES in a large cohort of Chinese patients diagnosed with obesity or diabetes. Our study is also the first to construct a panel covering genes causative for both obesity and diabetes, since these two metabolic disorders often accompany each other and are sometimes difficult to distinguish. In this study, 25 patients were initially diagnosed with both obesity and diabetes based on their clinical phenotypes, and clinicians experienced difficulties in accurately diagnosing these patients. Through genetic testing, a pathogenic MODY-related variant was identified in some of these patients, and they were finally diagnosed with MODY. In others, pathogenic obesity-causing variants were identified leading to the final diagnosis of monogenic obesity complicated by T2DM. Moreover, some patients harboured pathogenic variants associated with both obesity and diabetes, whereas some participants did not carry any pathogenic variant.

Over 70% of genes detected in this study was autosomal dominant for the reason that monogenic diseases such as monogenic obesity and MODY were mainly autosomal dominant inherited. Among all obesity-related genes in the targeted panel, *MC4R* most frequently expressed variations, which is in accordance with the findings of previous reports (Kleinendorst et al., 2018). In four patients, five pathogenic *MC4R* variants were identified, of which two have been reported in the ExAC database, two were novel in East Asians, and one has never been described in any published literature. Unlike shown in previous studies that variations in *HNF1A* were the most common cause of MODY in Europe, North America, and Asia (Hattersley, 1998), it was not the most frequent cause in our study, which identified three pathogenic variants. Likewise, other common MODY-related genes such as *HNF1B* and *GCK* had two and three pathogenic variants, respectively. Although ABCCB-MODY is considered rare (Firdous et al., 2018), our results were unexpected in that the *ABCCB* gene had the largest number of six pathogenic variants, possibly because *ABCC8* is a large 39-exon gene that does not allow routine Sanger sequencing. By contrast, high-throughput next-generation sequencing facilitated *ABCC8* testing and led to this unpredicted result. Besides, these inconsistent results may also be due to differences in sex distribution, sample size, and clinical criteria for genetic screening.

The high concordance of detected variants demonstrated the reliability of both methods. However, coverage was significantly higher in the targeted panel, and WES was more likely to miss variants or produce false-positive results due to insufficient depth of coverage. Usually a standard depth (∼50×–60×) sequencing in peripheral samples (such as blood) is recommended for WES (Mirzaa et al., 2016). The mean coverage depth for WES in this study was 96.7×, which meant the quality of WES performed in our study was enough to make comparisons with targeted panel. Besides, panels produce a smaller data volume and thus save costs for data storage and analysis. Panels also offer a simpler workflow with a faster turnaround time for routine testing. Furthermore, the cost for panel sequencing in our study was ∼1/30 of that for WES. To be specific, WES cost for each sample was >100 pounds (>800 yuan), whereas targeted sequencing just costs an average of ∼5 pounds (∼40 yuan) for each patient. Different panels vary in the number of genes included, thus leading to a variability of costs. The exact cost for each sample depend on the number of samples tested and will further decrease with increasing sample number. Moreover, a targeted gene panel avoids incidental uncovering of a genetic variant associated with other diseases and therefore circumvents ensuing ethical conflicts. Accordingly, in cases where the clinical information is highly consistent with several candidate genes, a targeted panel is a reasonable first-line choice. Our conclusions were consistent with previous studies. Researchers found that targeted gene panel was a faster and more effective screening method compared with WES for diagnosing early-onset inflammatory bowel disease (Petersen et al., 2017), inherited retinal dystrophies (Wang et al., 2018), movement disorders (Montaut et al., 2018), and Mendelian diseases (Saudi Mendeliome Group, 2015).

However, targeted gene panel sequencing also has shortcomings. Since the genetic cause of most patients with severe obesity and diabetes remains elusive and more contributory genes are discovered, the panel established in this study is not comprehensive and might soon be out of date. By contrast, WES is not limited to the detection of known genes. When a new causative gene is identified, WES data are sufficient for re-analysis. The ACMG recommends WES to be applied in nonspecific cases or in cases where more specific genetic tests such as targeted gene panels have failed to provide a diagnosis (ACMG Board of Directors, 2012). Our study showed that the main challenge for a diagnosis based on sequencing data is the classification of a variant's pathogenicity. Considering complicated clinical phenotypes of patients, unpublished causative variants, and lack of functional data, the current understanding of genes and variants often limits the optimal classification. The results of this study, therefore, not only serve a diagnostic purpose but also facilitate further research. The variants classified as VUS will be further evaluated to determine their clinical relevance. Additionally, our study did not routinely perform genetic testing on family members; therefore, the interpretation of the pathogenicity of variants was more challenging, especially those variants with a known recessive inheritance mode.

Although sequencing technologies have been greatly improved, the major limitations of both targeted panel sequencing and WES remain constant. Neither can identify changes in noncoding regions, and not all targeted exons have sufficient depth of coverage, especially those with a high GC content. Furthermore, chromosomal deletions, large structural variants, rearrangements, or duplications are not easily detected (O'Donnell-Luria and Miller, 2016). Quite a few patients in this study did not have variants fitting their diagnosed disease despite their clinical data suggesting a genetic predisposition. Technically, WGS would overcome all the above limitations. Nonetheless, WGS is not yet widely available for clinical diagnosis because of its higher costs, the larger amount of data generated, and more complicated data processing. Therefore, the targeted gene panel is favoured for patients with specific phenotypes, whereas WES is preferred if a disease phenotype is nonspecific. However, if the targeted panel or WES yields negative results, WGS may be considered a secondary alternative.

In conclusion, targeted gene panel sequencing is a quick and less costly method compared to WES while generating data of higher quality for genes of interest. These characteristics make the use of targeted gene panels, particularly the panel presented in this study, a promising first-line solution for routine clinical diagnosis of monogenic obesity and diabetes. Subsequent WES can be an alternative method for unsolved cases. Our study helps to provide a more accurate clinical diagnosis of monogenic obesity and diabetes, subsequently paving the way for gene-based treatment and genetic counselling, which helps to move step-by-step towards precision medicine. However, further research in larger cohorts is needed in the future.

## Materials and methods

### Participants

A cohort of 481 Chinese patients diagnosed with suspected monogenic obesity or diabetes were recruited from Shanghai Sixth People's Hospital for sequencing between 2017 and 2022. All patients were evaluated by clinical endocrinologists. A total of 146 samples were sequenced using both the targeted gene panel and WES. The remaining 335 patients only received panel sequencing. This study was approved by the Institutional Review Board of the Shanghai Sixth People's Hospital, and all participants provided informed consent. Written informed consent was obtained from all patients or the legal guardians of minors and children.

### Design of the targeted gene panel

For the probe design of the targeted capture regions, we selected a total of 83 genes of interest where mutations had been reported to be causative for monogenic obesity or diabetes: *MC3R, MC4R, SIM1, BDNF, MCHR1, LEP, LEPR, PCSK1, NTRK2*, and *POMC* for monogenic obesity (Loos and Yeo, 2022); *BBS1, BBS2, ARL6, BBS4*, and *MKKS* for BBS (Florea et al., 2021); *GLIS3, PLAGL1, PTF1A, INSR*, and *TBC1D* for NDM (Laimon et al, 2021); *HNF4A, GCK, HNF1A, PDX1, HNF1B, NEUROD1, KLF11, CEL, PAX4, INS, BLK, ABCC8, KCNJ11*, and *APPL1* for MODY (Delvecchio et al., 2020); *AKT2, AGPAT2, BSCL2, CAV1, LMNA, PPARG*, and *ZMPSTE24* for lipodystrophy (Jéru, 2021); and some other associated genes for syndromic diabetes such as Wolfram syndrome, hemochromatosis, and Alström syndrome (Zou et al., 2018; Meola, 2020; Kristan et al., 2021; Sayed and Nabi, 2021; Zhang et al., 2021; Prida et al., 2022). These genes can cause the disease alone or in combination. Detailed information on these 83 genes is listed in Supplementary Table S4.

### DNA library preparation and targeted sequencing

Genomic DNA was isolated from peripheral blood samples. The concentration and purity of the DNA samples were measured using a NanoDrop 2000. A total of 83 representative capture probes of metabolic disease-related genes were designed using the Agilent SureDesign online tool. The design parameters were: tiling density: 2×; masking: moderately stringent; boosting: balanced; extension into repeats: 20; strand: sense. The capture probe covers an area of 476.147 kb, including all target genes. To obtain the connection product, the sample DNA segments were connected with sticky ends to the connector. Then, 83 gene probes were hybridized and captured to obtain the target fragment. The purified target fragment was amplified by super multiplex polymerase chain reaction (PCR), and the amplified products obtained after separation and purification constituted a high-throughput library of genes related to metabolic diseases. Finally, based on the Ion Torrent sequencing platform, the amplification products of the target region were sequenced with high throughput, and all exon regions of the 83 target genes were detected simultaneously in one sequencing reaction. According to the flux of different chips, the number of detection samples can be flexibly adjusted on the premise of ensuring the average sequencing depth of ∼500×.

### WES

DNA was treated with NimbleGen SeqCap EZ Exome v3.0 capture reagent (capture target: 64 Mb, Roche) and TruSeq DNA sample preparation kit (Illumina) to construct the sequencing library. According to the manufacturerʼs instructions, the library was sequenced on the Illumina Novaseq 6000 platform with paired-end 150-bp reads.

### High-throughput DNA sequencing data analysis

The Burrows–Wheeler Aligner (bwa-mem, version 0.7.17-r1188) was used to align the raw sequences to the human reference genome build hg19/GRCh37. SAMtools (version 1.9) was applied to convert the alignment sam file to a sorted, indexed bam file. For WES, we used Picard to remove the PCR duplicates, followed by the base quality score recalibration with GATK BaseRecalibrator (version 3.7). Finally, GATK HaplotypeCaller (version 3.7) was employed to jointly call germline variants in all samples. The raw multi-sample vcf file was annotated using Annovar.

### Variant filtering and pathogenicity assessment

Variants were selected through a sequential process (Supplementary Figure S2). We selected non-synonymous and loss-of-function variants, including missense, stop-gain/loss, splice-site, and small frameshift insertion/deletion (InDel). We initially focused on exonic regions and filtered out synonymous variants and non-frameshift InDels at the protein level. To remove polymorphic loci, we applied a maximum threshold of 1% for allele frequency in this study cohort, 2% for MAF in all populations, and 1% MAF for the East Asian population in the oneKG (Auton et al., 2015), ExAC (Lek et al., 2016), gnomAD (Karczewski et al., 2020), and esp6500 (Fu et al., 2013) databases. The remaining variants with CADD scores >20 and DANN scores >0.98 were considered candidates for altering the protein structure. Genomic positions with a GERP score >2.0 were considered to be conserved. We also referred to additional public tools that predict damaging or deleterious variants, such as MetaSVM, Polyphen2, SIFT, and M-CAP. Taking the mode of inheritance into account, heterozygous mutations of genes with a known dominant inheritance can be causative, but for genes with known recessive or unknown inheritance, we needed to screen for homozygous mutations or compound-heterozygous variants. Based on all the above results, as well as the clinical information about the patients, all selected variants were classified according to the ACMG guidelines (Richards et al., 2015). Only the variants identified as ‘pathogenic’, ‘likely pathogenic’, and ‘VUS’ were further discussed in our study. Those classified as benign or likely benign were not included.

### Validation by sanger sequencing

Remaining variants identified as disease-causing were further confirmed by Sanger sequencing. The coding regions were amplified by a standard PCR protocol. Then, the PCR products were depurated and sequenced directly using the 3130 Genetic Analyzer (Applied Biosystems). PCR primers as well as PCR conditions are available upon request.

## Supplementary Material

mjad040_Supplemental_File

## Notes

**Author contributions:** C.H. designed the study. R.Z. conducted the experiments. Hairong Yu, Haoyong Yu, Y.B., H.Z., W.J., and C.H. analysed and interpreted the data, drafted, and revised the manuscript. D.P., D.Y., and Y.G. contributed to the sample and data collection. All authors have read and approved the final version of the manuscript. H.Z. and C.H. are the guarantors of this study.
