# Supplementary material for: Targeted gene panel provides advantages over whole-exome sequencing for diagnosing obesity and diabetes mellitus
Source: J Mol Cell Biol. 2023 Jun 16;15(6):mjad040. doi: 10.1093/jmcb/mjad040 (PMC10847719; doi:10.1093/jmcb/mjad040)
Supplement: mjad040_Supplemental_File [file mjad040_supplemental_file.pdf]

Supplementary Figures

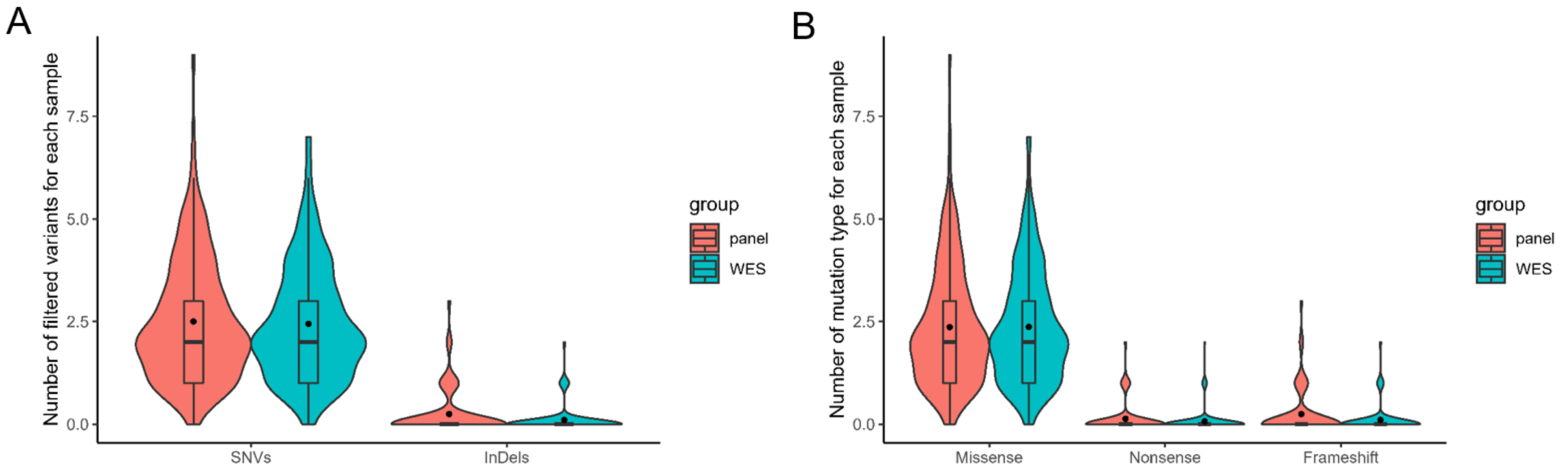

Supplementary Figure S1 Number of variants for each sample in panel and WES after filtering. (**A**) SNVs and InDels for each sample. (**B**) Further classification of SNVs (missense and nonsense variants) and InDels (frameshift variants) for each sample. SNV, single nucleotide variant; InDel, insertion or deletion.

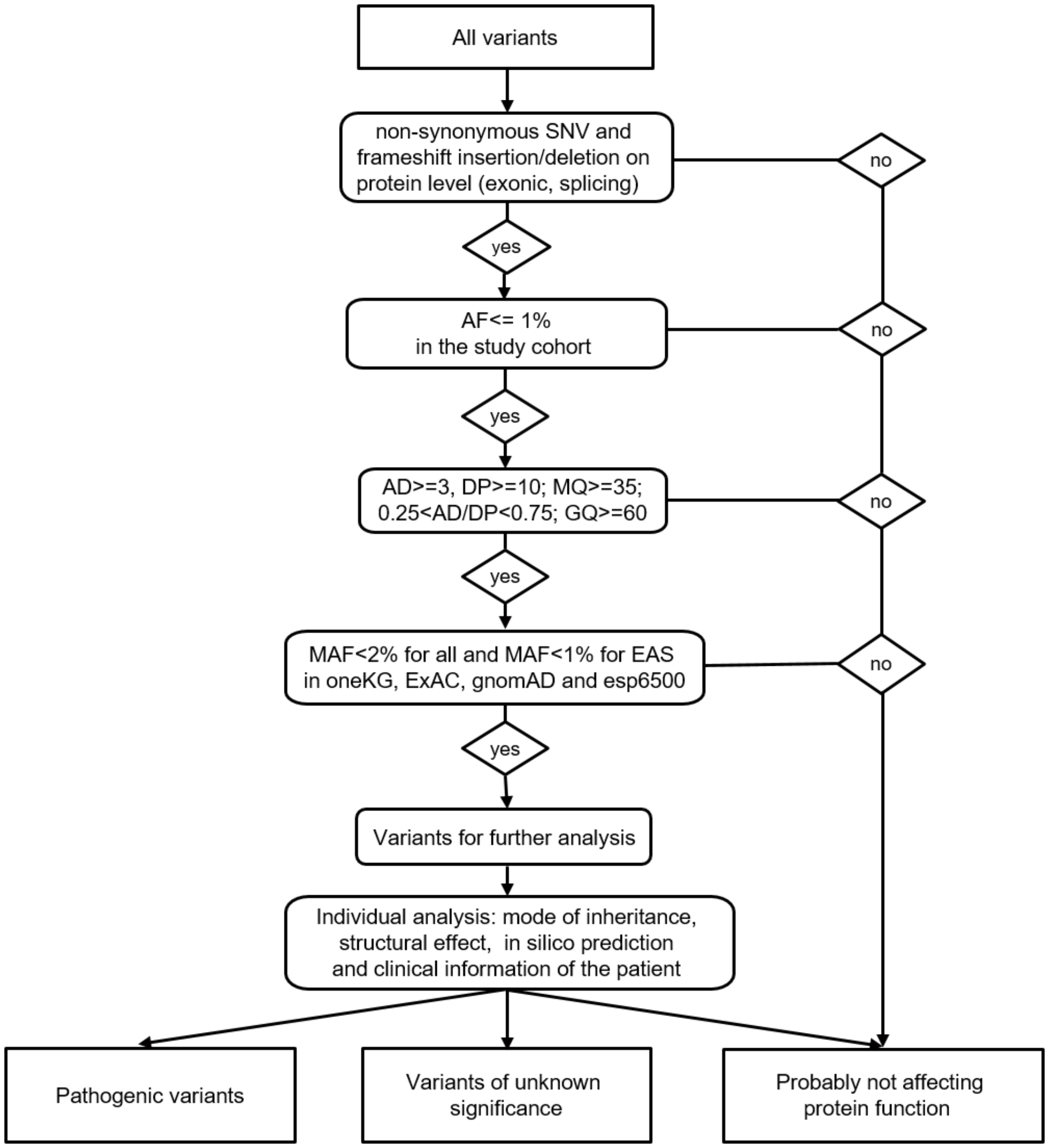

Supplementary Figure S2 Flowchart for filtering and classification of detected variants.

Supplementary Tables

Supplementary Table S1 Characteristics of patients.

	Panel + WES	Panel only
Total number	146	335
Male	68 (46.5%)	96 (28.7%)
Female	78 (53.4%)	239 (71.3%)
Age range (years)	2–70	7–63
Median age (years)	28	30.5
Pediatric (<18 years)	19 (13.0%)	11 (3.3%)
Adult (⩾18 years)	127 (87.0%)	324 (96.7%)
Obesity	83 (56.8%)	240 (71.6%)
Obesity (with diabetes)	19 (13.0%)	60 (17.9%)
MODY	30 (20.5%)	35 (10.4%)
NDM	7 (4.8%)	0
Syndromic diabetes	7 (4.8%)	0

MODY, maturity-onset diabetes of the young; NDM, neonatal diabetes mellitus; WES, whole-exome sequencing.

Supplementary Table S2 Variants identified only by targeted gene panel.

Patient no.	Age (years)	Sex[a]	BMI (kg/m^2)	Diagnosis	Gene	Chr	Position	SNP	Ref	Alt	Effect	Inheritance	Pathogenic prediction	GERP	AA change	MAF in EAS[b]	Variant classification
30	31	2	42.6	MODY	*WFS1*	4	6303542	rs200672755	G	A	missense	AD	damaging	5.49	NM_001145853:exon8:c.G2020A:p.G674R	0.02%	VUS
73		1		OB	*TBC1D4*	13	75898423		GC	G	frameshift	AD			NM_014832:exon11:c.2147delG:p.S716Tfs*43	Novel	LP
75	35	2	40.1	OB	*HNF4A*	20	43019284	rs780566668	C	T	missense	AD	damaging	4.15	NM_001287182:exon2:c.C5T:p.S2L	0	VUS

Chr, chromosome; SNP, single-nucleotide polymorphism; Ref, reference allele; Alt, alternative/variant allele; OB, obesity; MODY, maturity-onset diabetes of the young; AD, autosomal dominant; LP, likely pathogenic; VUS, variant of uncertain significance.

[a] 1 represents male, 2 represents female.

[b] MAF in East Asian population was referred to in ExAC database.

Supplementary Table S3 Novel variants detected by WES.

Patient no.	Age (years)	Sex[a]	BMI (kg/m^2)	Diagnosis	Gene	Chr	Position	SNP	Ref	Alt	Effect	Pathogenic prediction	GERP	AA change	MAF in EAS[b]	Variant classification
77	25	1	50.3	OB	*PRDM16*	1	3329036	rs752903093	C	T	missense	damaging	4.41	NM_022114:exon9:c.C2275T:p.R759W	0.08%	P
119	24	2	35.2	OB	*PRDM16*	1	3321424	rs748880850	C	T	missense	damaging	4.63	NM_022114:exon7:c.C1006T:p.R336C	0	P
120		1		OB	*KSR2*	12	117993048	rs754867135	G	T	missense	damaging	5.1	NM_173598:exon9:c.C1357A:p.P453T	0	P

Chr, chromosome; SNP, single-nucleotide polymorphism; Ref, reference allele; Alt, alternative/variant allele; OB, obesity; P, pathogenic.

[a] 1 represents male, 2 represents female

[b] MAF in East Asian population was referred to in ExAC database.

Supplementary Table S4 Genes included in the targeted gene panel, associated clinical syndromes and mode of inheritance.

Gene	Location	Clinical syndrome	Inheriance pattern	pLI	pHaplo
BBS1	11q13.2	Bardet-Biedl syndrome 1	AD/AR	0	0.05
BBS2	16q13	Bardet-Biedl syndrome 2	AR	0	0.27
ARL6	3q11.2	Bardet-Biedl syndrome 3	AR	0.01	0.09
BBS4	15q24.1	Bardet-Biedl syndrome 4	AR	0	0.41
BBS5	2q31.1	Bardet-Biedl syndrome 5	AR	0	0.1
MKKS	20p12.2	Bardet-Biedl syndrome 6	AR	0	0.65
BBS7	4q27	Bardet-Biedl syndrome 7	AR	0	0.29
TTC8	14q31.3	Bardet-Biedl syndrome 8	AR	0	0.66
BBS9	7p14.3	Bardet-Biedl syndrome 9	AR	0	0.47
BBS10	12q21.2	Bardet-Biedl syndrome 10	AR	.	0.26
TRIM32	9q33.1	Bardet-Biedl syndrome 11	AR	0	0.36
BBS12	4q27	Bardet-Biedl syndrome 12	AR	0	0.11
MKS1	17q22	Bardet-Biedl syndrome 13	AR	0	0.36
CEP290	12q21.32	Bardet-Biedl syndrome 14	AR	0	0.63
AKT2	19q13.2	Lipodystrophy; severe insulin resistance	AD	0.64	0.76
AGPAT2	9q34.3	Lipodystrophy, congenital generalized, type 1	AR	0	0.37
BSCL2	11q12.3	Lipodystrophy, congenital generalized, type 2	AD/AR	0	0.11
CAV1	7q31.2	Lipodystrophy, familial partial, type 7	AD	0.01	0.88
LMNA	1q22	Lipodystrophy, familial partial, type 2	AD	1	0.72
PPARG	3p25.2	Lipodystrophy; Insulin resistance, severe, digenic	AD	0.03	0.96
ZMPSTE24	1p34.2	Lipodystrophy	AR	0	0.57
DMPK	19q13.32	Myotonic dystrophy 1	AD	0.05	0.23
CNBP	3q21.3	Myotonic dystrophy 2	AD	0.96	0.85
BDNF	9q21.33	Obesity, hyperphagia, and developmental delay	AD	0.77	0.86
MC3R	20q13.2	Obesity, severe	AD	0	0.21
MC4R	18q21.32	Obesity	AD/AR	0	0.14
MCHR1	22q13.2	Obesity	AD	0	0.32
LEP	7q32.1	Obesity, morbid, due to leptin deficiency	AR	0.46	0.52
LEPR	1p31.3	Obesity, morbid, due to leptin receptor deficiency	AR	0.99	0.91
NTRK2	9q21.33	Obesity, hyperphagia, and developmental delay	AD	1	0.93

Gene	Location	Clinical syndrome	Inheriance pattern	pLI	pHaplo
PCSK1	5q15	Obesity with impaired prohormone processing	AD	0	0.17
POMC	2p23.3	Obesity, early-onset	AD/AR	0	0.49
SIM1	6q16.3	Obesity	AD	1	0.93
HNF4A	20q13.12	MODY1	AD	0.38	0.55
GCK	7p13	MODY2; Neonatal diabetes; Hyperproinsulinemia	AD	0.24	0.71
HNF1A	12q24.31	MODY3	AD	0.96	0.92
PDX1	13q12.2	MODY4, T2D	AD	0.02	0.71
HNF1B	17q12	MODY5; Neonatal diabetes	AD	.	0.96
NEUROD1	2q31.3	MODY6	AD	0.77	0.92
KLF11	2p25.1	MODY7	AD	0	0.8
CEL	9q34.13	MODY8	AD	0	0.47
PAX4	7q32.1	MODY9; Diabetes mellitus, type 2	AD	0	0.45
INS	11p15.5	MODY10; Permanent neonatal diabetes; Hyperproinsulinemia	AD	0.3	0.79
BLK	8p23.1	MODY11	AD	0	0.5
ABCC8	11p15.1	MODY12; Diabetes mellitus, noninsulin-dependent	AD	0	0.59
KCNJ11	11p15.1	MODY13; Permanent neonatal diabetes; Hyperproinsulinemia	AD	0.01	0.5
APPL1	3p14.3	MODY14; insulin resistance	AD	0.8	0.85
FOXA2	20p11.21	MODY	AD	0.74	0.96
ISL1	5q11.1	MODY	AD	0.87	0.95
MAPK8IP1	11p11.2	MODY; Diabetes mellitus, noninsulin-dependent	AD	1	0.77
NEUROD4	12q13.2	MODY	AD	0.1	0.12
PAX6	11p13	MODY	AD	1	0.97
TGM2	20q11.23	MODY; Huntington disease	AD	0	0.55
FOXP3	Xp11.23	Neonatal diabetes mellitus; X-linked multiple endocrine disease	XLR	0.99	.
GLIS3	9p24.2	Neonatal diabetes mellitus; diabetes	AR	0	0.93
PLAGL1	6q24.2	Neonatal diabetes mellitus	AD	0.98	0.98
PTF1A	10p12.2	Neonatal diabetes mellitus	AR	0.17	0.97
INSR	19p13.2	Diabetes mellitus, insulin-resistant; Hyperinsulinemic hypoglycemia	AD	0	0.91

Gene	Location	Clinical syndrome	Inheriance pattern	pLI	pHaplo
TBC1D4	13q22.2	Diabetes mellitus, noninsulin-dependent, obesity	AD	0	0.8
WFS1	4p16.1	Wolfram syndrome	AD	0	0.27
PPP1R3A	7q31.1	Severe insulin resistance syndrome, digenic	AD	0	0.13
CHN2	7p14.3	Severe insulin resistance syndrome	AD	0	0.87
CFTR	7q31.2	Chronic pancreatitis	AD	0	0.12
CTRC	1p36.21	Chronic pancreatitis	AD	0	0.42
PRSS1	7q34	Chronic pancreatitis	AD	0	0.26
PRSS2	7q34	Chronic pancreatitis	AD	.	.
SPINK1	5q32	Chronic pancreatitis	AD	0.31	0.52
CP	3q24-q25.1	Hemosiderosis, systemic, due to aceruloplasminemia	AR	0	0.4
HFE	6p22.2	Hemochromatosis	AR	0	0.21
HFE2	1q21.1	Hemochromatosis, type 2A	AR	0	.
HAMP	19q13.12	Hemochromatosis, type 2B	AR	0.01	0.37
TFR2	7q22.1	Hemochromatosis, type 3	AR	0	0.38
SLC40A1	2q32.2	Hemochromatosis, type 4	AD	0.99	0.96
ALMS1	2p13.1	Alstrom syndrome	AR	.	0.09
AIRE	21q22.3	Autoimmune polyendocrinopathy syndrome, type I	AD/AR	0	0.32
EIF2AK3	2p11.2	Wolcott-Rallison syndrome	AR	0	0.68
FXN	9q21.11	Friedreich ataxia	AR	0.11	0.2
GLUD1	10q23.2	Hyperinsulinism-hyperammonemia syndrome	AD	0.01	0.74
HADH	4q25	Hyperinsulinemic hypoglycemia, familial, 4	AR	0.01	0.56
HTT	4p16.3	Huntington disease	AD	1	1
TRIM37	17q22	Mulibrey nanism	AR	0	0.36
SLC19A2	1q24.2	Thiamine-responsive megaloblastic anemia syndrome	AR	0	0.43
WRN	8p12	Werner syndrome	AR	0	0.79

AR, autosomal recessive; AD, autosomal dominant; XLR, X-linked recessive.

pLI, probability of loss-of-function intolerance. Values range from 0 to 1. Genes with larger values (closer to 1) are more intolerant of mutations.

pHaplo, predicted probability of Haplo insufficiency. pHaplo scores ≥0.86 indicates a high possibility of haplo insufficiency.
